# Associations between Avocado Consumption and Diet Quality, Dietary Intake, Measures of Obesity and Body Composition in Adolescents: The Teen Food and Development Study

**DOI:** 10.3390/nu13124489

**Published:** 2021-12-15

**Authors:** Gina Segovia-Siapco, Michael Paalani, Keiji Oda, Peter Pribis, Joan Sabaté

**Affiliations:** 1 School of Public Health, Loma Linda University, 24951 No. Circle Drive, Loma Linda, CA 92350, USA; mpaalani@llu.edu (M.P.); koda@llu.edu (K.O.); jsabate@llu.edu (J.S.); 2 Department of Individual, Family & Community Education, Nutrition and Dietetics Program, College of Education & Human Sciences, University of New Mexico, Albuquerque, NM 87131, USA; pribis@unm.edu

**Keywords:** diet quality index-international, shortfall nutrients, obesity, adolescence, children, vegetarian, plant-based, adequacy, moderation

## Abstract

Avocado is a nutrient-rich food that has been shown to benefit the health and diet quality of adults. In this paper, we examined if habitual intake of avocado among adolescents is associated with their diet quality, food and nutrient intake, and measures of obesity and body composition. Participants aged 12–18 years old (*n* = 534) from selected public and Adventist middle-high schools reported their dietary intake in a web-based food frequency questionnaire (FFQ); anthropometrics were measured during school visits. Diet quality (DQI-I) and avocado intake were calculated from the FFQ; BMI z-scores (BMIz), waist-to-height ratio (WHtR), and fat mass (FM), fat-free mass (FFM), and %body fat (%BF) were determined from the anthropometric data. Compared to non-consumers, avocado consumers had significantly higher covariate-adjusted mean scores on total DQI-I (68.3 vs. 64.6) and energy-adjusted mean scores on variety (18.8 vs. 18.0) and adequacy (36.4 vs. 33.4). Avocado consumption was significantly associated with DQI-I components adequacy (β [SE] = 0.11 [0.03]) and moderation (β [SE] = 0.06 [0.03]) but not with BMIz, WHtR, FM, FFM, and %BF. Mean intakes of fruits, vegetables, and plant protein foods, total and vegetable proteins, dietary fiber, retinol, vitamin C, calcium, magnesium, iron, and potassium were significantly higher for avocado consumers; saturated and trans fats intakes were significantly higher for non-consumers. In our adolescent population, avocado consumption was associated with higher diet quality and intake of plant-based foods and shortfall nutrients, but not with measures of obesity and body composition. Studies are needed to determine the optimal amount of avocado that would confer health benefits during adolescence.

## 1. Introduction

The increase in independence during adolescence has been shown to influence dietary habits and food preferences of youth. Late snacking [1], meal-skipping, and consumption of energy-dense but nutrient-poor convenience foods and sweetened beverages characterize most adolescents worldwide [2,3]. Poor diet quality as indicated by low or very low consumption of nutritionally healthy foods is associated with being overweight or obese among adolescents [4]. Trend analysis of diet quality using National Health and Nutrition Examination Survey (NHANES) 2003–2018 data shows that, although there had been improvements in the quality of foods consumed coming from schools, grocery stores, and restaurants, children 5–19 years of age remain to have poor diet quality compared to adults [5]. Unhealthful eating practices during childhood and adolescence could extend into adulthood [6,7]. Therefore, educating teenagers to improve their dietary habits can provide significant public health benefits on a local, national, and global scale.

Incorporation of certain foods and/or food groups into the diet had been shown to improve or exhibit better diet quality. This may be due to what is usually eaten alongside those foods, or by displacing certain less healthy foods with healthier alternatives [8,9,10]. Specifically, avocados have high nutritional value, since they are rich in vitamins, minerals, fiber, and phytochemicals [11]. Incorporating avocados into the diet has been associated with health-related benefits, by lowering the risk for conditions such as metabolic syndrome and cardiovascular disease [12,13], and reducing the risk for becoming overweight or obese due to increased satiety and reduced hunger [14]. However, although optimal nutrition can have beneficial effects on health outcomes, studies that analyzed the relationship between avocado intake and diet or nutritional quality are scant [15] despite a substantial number of studies on avocado and its health benefits [12,13,14].

As a medium-energy nutrient-rich food, avocados have the potential to improve diet quality. A study among adults showed that compared to non-consumers, those who consume avocado have better diet quality and nutrient intakes, and lower cardiometabolic risk factors [15]. So far, no investigation on these same variables has been carried out among adolescents. Thus, we determined if habitual avocado intake among adolescents is associated with diet quality, food and nutrient intake, and anthropometric measures of general and central adiposity and body composition.

## 2. Materials and Methods

### 2.1. Study Design and Participants

The Teen Food and Development Study is a cross-sectional study that was conceived to determine how consumption of certain foods among adolescents are associated with their physical growth and pubertal development [16]. Recruitment of the study population had been described elsewhere [17]. Briefly, adolescents aged 12–18 years old (*n* = 601) were recruited via convenience sampling from 8 selected public and Adventist middle and high schools near Adventist universities in southern California and Michigan, when the study was conducted from February 2012 to May 2013. Participants responded to a web-based questionnaire especially designed for the study and attended clinics held at their schools for anthropometric measurements. Participants provided their assent together with consent from their parents before completing the web-based questionnaire, which comprised sections on dietary intake (food frequency questionnaire), lifestyle, physical growth and pubertal development, and demographics.

### 2.2. Measurements

#### 2.2.1. Dietary and Avocado Intake

The web-based questionnaire used in the study included a 151-item semi-quantitative food frequency questionnaire (FFQ) that had been validated for its nutrient intake estimates [18]. Briefly, the FFQ includes 32 convenience foods, 29 protein-rich foods, 17 breads/grains/cereals, 21 vegetable and fruit items, 10 dairy products, 24 beverages, 11 snack/sweets, and 7 legumes/soups. In the FFQ, frequency of intake for fixed portion sizes of the food items are categorized as never/rarely, one to three times per month, once per week, two to four times per week, five to six times per week, once per day, two to three times per day, and four or more times per day. For this study, food groupings used in the Diet Quality Index-International (DQI-I) scoring scheme were created and measured in servings/day. The nutrient profile of the FFQ foods were derived from the Nutrition Data System for Research (NDSR) software version 2012, developed by the Nutrition Coordinating Center of the University of Minnesota, Minneapolis, MN. Intake of nutrients used in the DQI-I scoring scheme were computed from these nutrient profiles and the frequency of intake of each food item per day using the product-sum method.

The FFQ includes an item on avocado/guacamole and other mixed foods known to contain avocado (e.g., sushi). Using the product-sum method, total avocado intake was computed. Considering the very skewed distribution of total avocado intake and a lack of necessary variation among avocado consumers, participants were grouped into two: avocado non-consumers (zero avocado intake) and avocado consumers (intake of any amount of avocado).

#### 2.2.2. Diet Quality

Diet quality was measured using the Diet Quality Index-International (DQI-I), one of the many existing tools used to measure diet quality. The DQI-I has four major components: (1) variety across food groups and within the protein food group (accounts for 20 points of the DQI-I); (2) adequacy of intake of eight food groups and nutrients based on existing dietary recommendations (40 points); (3) moderation in intake of foods and nutrients known to be related to chronic diseases (30 points); and (4) overall balance in the intake of macronutrients and fatty acids based on dietary recommendations (10 points) [19]. Different population groups have used the DQI-I and modified the tool to accommodate existing dietary guidelines and/or recommendations [20,21,22,23,24]. The diet quality of child and adolescent populations have also been assessed utilizing this tool [20,24,25,26,27].

For our adolescent population, the Dietary Guidelines for Americans 2020–2025 [28] were used to determine the recommended food group intakes, while the Dietary Reference Intakes [29] were used to determine the recommended nutrient intakes for male and female adolescents. Details on how the DQI-I components were scored are shown in Table 1.

#### 2.2.3. Indicators of Obesity and Body Composition

Trained research assistants measured weight, height, waist and hip circumferences, and body composition during school visits. Height was measured using a stadiometer (Seca Portable™, Chino, CA, USA) while weight and body composition (fat mass, fat-free mass, %body fat) were measured with a bioelectric impedance analysis scale (TANITA™, model TBF-300A, Arlington Heights, IL, USA). Waist and hip circumferences were measured with a non-stretchable tape. All measurements were carried out twice to the nearest tenth of the unit measure, and the average of the two measurements was used. Body mass index (BMI) was calculated from the height and weight measurements, then age-and-gender-specific indicators of general body obesity (BMI z-score, BMIz) and central adiposity (waist-to-height ratio, WHtR) appropriate for the adolescent population [30,31] were computed. In the web-based questionnaire, participants were also asked to self-report their height, weight, waist and hip circumferences corresponding to instructions on how to measure themselves.

### 2.3. Covariates

The web-based questionnaire includes sections on demographics (age, gender, ethnicity, educational levels of mother and father), lifestyle (sleep, vigorous physical activity), and physical growth (self-reported weight, height, waist and hip circumferences). Father’s educational level was used as the surrogate for socio-economic status.

### 2.4. Data Analysis

Of the 601 participants, 11 were excluded from the analysis due to non-response to several items in the web-based questionnaire. An additional 56 participants were also excluded due to implausible energy intake (female: <600 or >3700 kcal; male: <800 or >4500 kcal). Thus, the analytical dataset was composed of 534 participants. This sample size would provide enough power (80%) to find a small effect size of 0.22 at an alpha level of 0.05.

Descriptive statistics, comparison tests, and general linear modeling were used to analyze the data. The dietary variables (intakes of avocado, food groups, and nutrients) and DQI-I total and component scores, and anthropometric variables with non-normal distributions were normalized using the appropriate transformations. Nutrient and food group variables were energy-adjusted using the residual method.

### 2.5. Comparison of Diet Quality and Dietary Intake

To examine if avocado consumers and non-consumers significantly differed in their diet quality and intake of selected food groups and nutrients, appropriate comparison tests and linear modelling were used. The linear model adjusted for: gender, age, study site, father’s education, ethnicity, hours of sleep, physical activity (min/day), and vegetarian status. To deal with missing values, multiple imputation was performed by chained equations using “mice” package version 3.13.0 [32], assuming missing at random. Five imputation datasets were generated by predictive mean matching. Twenty iterations were run before each imputation. For each imputation dataset, beta coefficients were estimated based on the model described above, and these estimates were pooled using Rubin’s rule. Estimated marginal means (with 95% confidence intervals) for DQI-I scores were then obtained for avocado groups, adjusting for all the other variables in the model. Regression assumptions were examined by visually inspecting residual plots (before multiple imputation). Estimated marginal means (EMM) and mean differences, or back transformed EMM with back transformed mean ratios were reported.

### 2.6. Association between Avocado Intake and Anthropometric Indicators of Health

A linear model was fitted for each of the following anthropometric measures of health: BMIz, WHtR, fat mass (FM), fat-free mass (FFM), and % body fat (%BF). Covariates controlled for were age, gender (gender stratification was carried out for body composition—FM, FFM, and %BF—since it is known that fat mass and fat-free mass differ between males and females), square term for centered age, study site, father’s educational level (used as surrogate of socio-economic status), ethnicity, sleep hours, physical activity, vegetarian status, total energy (100 kcal/day), and energy-adjusted animal protein intake (10 g/d). Model assumptions were examined using residual plots. The linearity assumption of energy-adjusted avocado intake was assessed using partial regression plots. Multicollinearity was assessed by the variance inflation factor, and no multicollinearity was found. To address missing values in covariates, imputation procedures were run as described earlier.

### 2.7. Determinants of Avocado Intake

Logistic regression analysis was conducted to determine which DQI-I components and demographic variables were associated with avocado intake. Food groups associated with avocado intake were determined using correlation analysis.

## 3. Results

Table 2 shows the demographic and lifestyle characteristics of all participants, and when grouped according to avocado consumption. About 60% of the study population were avocado consumers with a mean intake of 62 g per week, which is approximately two-thirds of a medium size avocado. Avocado consumers were significantly older, more likely to be from California, with higher socio-economic status, more likely to be Hispanic, and less likely to be African/African American.

### 3.1. Diet Quality and Anthropometric Health Indicators of Avocado Consumers and Non-Consumers

A comparison of the diet quality scores shows significantly higher scores for avocado consumers than non-consumers on total DQI-I and its components, variety and adequacy, but no significant differences for the components, moderation and overall balance (see Table 3). It is notable that the scores for variety and adequacy were relatively closer to the maximum possible scores (18.0 and 18.8 out of 20 points for non-consumers and consumers, respectively, for variety; and, 33.4 and 36.4 out of 40 points for non-consumers and consumers, respectively, for adequacy) compared to scores for moderation (10.2 and 10.6 out of 30 points for non-consumers and consumers, respectively) and overall balance (1.3 and 1.7 out of 10 for non-consumers and consumers, respectively). Scores for overall food group variety of intake and variety in protein food sources were significantly higher among avocado consumers, as well as adequacy scores for intake of vegetables, fruits, fiber, vitamin C, and calcium. Although scores for moderation were low for both groups, consumers have significantly higher scores on moderation in total fat and saturated fat intakes. However, their moderation score for sodium intake was significantly lower than non-consumers. Scores for balance in macronutrient ratio and fatty acid ratio intakes were not different between the groups, which scored low on both (see Table 3).

**Figure 1:**
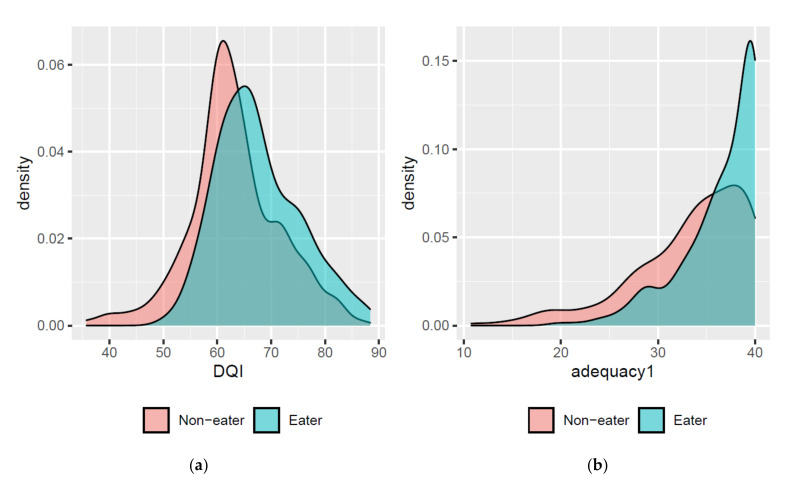
(**a**) Distribution densities of the DQI scores indicate a “shift” to an improved diet quality for avocado consumers compared to non-consumers among adolescents. (**b**) Although both groups seem to have adequate intake, the adequacy of intake is greater among avocado consumers.

Results show that there were no significant differences between avocado consumers and non-consumers on any of the anthropometric indicators of health (BMIz for general body adiposity, WHtR for central adiposity) and body composition (FFM, FM, and %BF) (data not shown). Table 4 shows that the estimated marginal means for DQI-I total score increased slightly and remained significantly different between consumers and non-consumers (68.3 vs. 64.9, respectively, *p* < 0.0001) when controlled for covariates. The nature of the distributions of the DQI-I component scores restricted the ability to control for confounders, since normalization of the distributions could not be achieved. Avocado consumers have significantly higher values for DQI-I total and adequacy than non-consumers. See the density plot by avocado intake in Figure 1. Table 4 also shows that adjustment for confounders did not alter the previous finding that avocado consumers and non-consumers have similar anthropometric health parameters.

### 3.2. Dietary Intake of Avocado Consumers and Non-Consumers

Mean intakes of fruits, vegetables and plant protein foods were significantly higher for avocado consumers compared to non-consumers (Table 5). Intakes of several nutrients (total and vegetable proteins, dietary fiber, retinol equivalents, vitamin C, calcium, magnesium, iron and potassium) were also significantly higher for consumers. Nutrients associated with chronic disease, trans fats and SFA, were both consumed significantly higher by the avocado non-consumers. However, consumers have significantly higher intake of sodium (~119 mg/day more) compared to their counterparts.

### 3.3. Determinants of Avocado Intake

**Figure 2:**
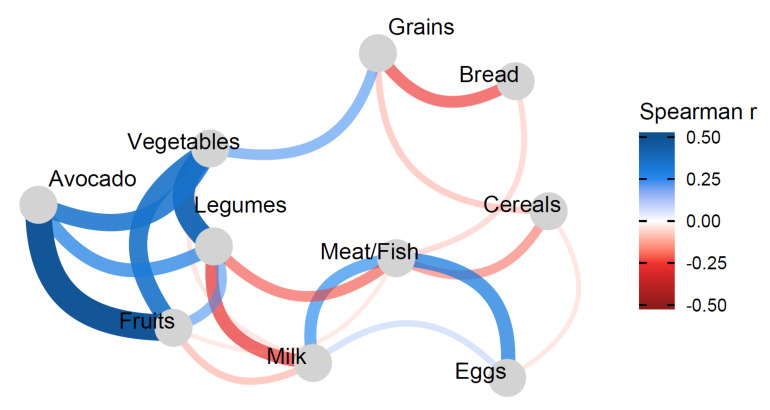
Correlation network showing the associations between avocado intake and intake of other foods. Blue represents positive while red represents inverse correlations. For examples, avocado intake is highly positively correlated with intake of fruits and moderately positively correlated with intake of vegetables and legumes, whereas legumes is strongly positively correlated with vegetables and positively moderately correlated with fruits, but moderately negatively correlated with intakes of milk and meat/fish; grains intake is weakly positively correlated with intake of vegetables but moderately negatively correlated with intake of bread and weakly negatively correlated with cereal intake, and so forth.

Table 6 shows that the DQI-I components adequacy and moderation were significantly positively associated with avocado intake. In addition, being a Hispanic or Asian compared to being a Caucasian was also positively associated with avocado intake. However, being a resident of Michigan compared to being a Californian as a participant, or having a high school education or less compared to graduate education, or being an African/African-American compared to being a Caucasian were negatively associated with avocado consumption. Figure 2 shows a correlation network of food groups that are most interrelated with each other. Avocado intake was most correlated with intakes of fruits, vegetables, and legumes.

## 4. Discussion

We determined if avocado intake is associated with diet quality, dietary intake, and anthropometric health parameters among adolescents. Our findings reveal that, compared to non-consumers, avocado consumers have better diet quality as indicated by higher scores on the DQI-I, have significantly higher intake of foods and nutrients known to be associated with better health, and lower intake of nutrients implicated in chronic diseases. However, we did not find associations between avocado intake and indicators of general body adiposity, central adiposity, and body composition. We found that of the four DQI-I components, adequacy and moderation were significantly associated with avocado intake; avocado intake is also most highly correlated with intake of fruits, legumes, and vegetables.

As far as we know, this is the first study among adolescents that investigated how avocado intake is associated with diet quality, dietary intake, and anthropometric indicators of health. Growth and development are at their peak during adolescence, so forms of malnutrition (whether through excessive caloric intake and/or nutrient-poor diets) can have detrimental effects on health. The quality of diet during this life stage is an important consideration when investigating the role of diet in the overall health of adolescents. We chose DQI-I as a measure of diet quality in this population since its components embody the basic healthy eating principles: variety, adequacy, moderation, and balance [28,29].

We found that in this population of adolescents, mean DQI-I scores were highest for variety (18.0 for non-consumers of avocado and 18.8 for consumers) and adequacy (33.4 for non-consumers of avocado and 36.4 for consumers). However, scores for moderation were mostly just a third of the maximum possible score of 30 points (10.2 for non-consumers of avocado and 10.6 for consumers), while scores for overall balance were just about a tenth of the maximum possible score of 10 points (1.3 for non-consumers of avocado and 1.7 for consumers). Although the diet quality scores for our population were relatively higher compared to what were found in other diet quality studies among young people, the pattern in DQI-I scores is similar to those in western countries (i.e., higher for variety and adequacy, but lower for moderation and overall balance). For instance, among Canadian children aged 8–10 years old whose diet was assessed using three 24-h diet recalls, overall DQI-I mean score was 57.9, mean score for variety was 15.5, 29.7 for adequacy, 11.9 for moderation, and 0.7 for overall balance [24]. A Mediterranean adaptation of the DQI-I to assess the diet quality of southern Spain youth aged 6–18 years old also have the same pattern: total DQI-I mean score was 56.3 points, 18.2 for variety, 26.4 for adequacy, 10.1 for moderation and 1.5 for overall balance [20]. On the other hand, a diet quality study among Sri Lankan adolescents [27] found moderation scores to be higher (ranged from 26.4 to 27.0 according to age groups) while scores for variety (ranged from 11.1 to 13.8) and adequacy (ranged from 9.6 to 14.8) were lower and thus, have a different DQI-I score pattern. Total DQI-I mean scores among the different age groups ranged from 48.6 to 56.9 and balance scores ranged from 0.81 to 2.00 [27].

The DQI-I was able to capture what aspect of the diet in the population contributes to the overall diet quality. Our results are consistent with study findings from other western adolescent populations [20,24] showing that in general, moderation in intake of nutrients and foods considered to have long-term detrimental health effects and overall balance in intake of macronutrients and specific types of fats are the aspects of diet that need more intervention in these populations. In developing economies where poverty impairs affordability and even accessibility of foods, variety and adequacy pose more challenge to diet quality. Limited evidence in adult populations also exhibit the same tendency for differences in DQI-I score patterns between developed and developing economies [19,33].

Of the four components of DQI-I, adequacy and moderation were significantly associated with avocado intake after controlling for potential confounders. We also found avocado to be moderately-to-highly correlated with intake of fruits, vegetables, and legumes. Although avocado is a monounsaturated fatty acid-rich fruit, we did not find avocado consumers to have a significantly higher consumption of MUFA. However, avocado consumers ate less saturated fatty acids and trans fats, possibly due to the type of foods more likely eaten with avocado by its consumers.

Our findings show that avocado consumers have a better dietary intake profile compared to their non-consuming counterparts. Intakes of total fat, saturated fatty acids, and trans fats (which are implicated in inflammation [34] and chronic diseases [35]) are higher among non-consumers of avocado. On the other hand, intakes of fruits, vegetables, vegetable protein, dietary fiber, vitamins A and C, calcium, magnesium, iron, and potassium (which include some of the shortfall nutrients for adolescents [36]) were higher among the avocado consumers. Avocado consumers have a dietary intake profile that reflects a better diet quality that is consistent with higher DQI-I scores for its components variety, adequacy (particularly for intake of vegetable and fruit groups, and fiber, protein, vitamin C, and calcium) and moderation, particularly for intake of total fat and saturated fat. However, the DQI-I score for moderation in sodium intake was significantly higher for the non-consumer group, which could be explained by the significantly lower intake of sodium in this group compared to the avocado consumers. Evidence shows that intakes of certain nutrient-rich or healthy foods are associated with or improve overall diet quality [8,9,10,37,38,39,40]. As a nutrient-rich food, intake of avocado had been shown to be associated with improved diet quality among US adults [15]. As shown in our study, avocado intake was moderately-to-highly correlated with legume, vegetable, and fruit intakes (shown in Figure 2) which could partially explain the better diet quality among its consumers.

Despite a better diet quality among avocado consumers, we did not find any association between avocado intake and anthropometric measures of general body adiposity (BMI z-scores), central adiposity (waist-to-height ratio), and body composition (fat mass, fat-free mass, and % body fat) in our adolescent population. Our sample size would have had enough power (80%) to find significant differences for a small effect size of 0.22 at an alpha level of 0.05. However, the differences between the avocado consumers and non-consumers were lower than the small effect size we anticipated. In a previous investigation among the Adventist Health Study 2 (AHS2) cohort on the effect of avocado intake on weight and body mass changes, avocado consumers have lower overweight/obesity odds compared to non-consumers [14]. In another study among US adults, a cross-sectional analysis of NHANES data shows avocado intake to be associated with lower risk of metabolic syndrome [15]. Perhaps, our results are different due in part to the lower mean intake of avocado in our adolescent population compared to the adult populations. Although about 60% of our participants reported to have consumed avocado, the energy-adjusted mean intake is 6.6 g daily compared to 70 g/d for the approximately 2% of the US study population that consume avocado [15] or the ~35 g/d by the high avocado consumers of the AHS2 cohort [14]. Similar investigations among children and adolescents are lacking, so it is still not known if there is indeed no association between avocado intake and young people’s anthropometric parameters. Further studies are needed to determine the role of avocado intake on young people’s health, and the optimal daily intake amount that would confer health benefits from avocado in the context of adiposity prevention among growing adolescents. However, diet quality has been shown to be associated with risk of metabolic syndrome [41], various anthropometric measures [24,42,43,44], health-related quality of life [45], and emotional and mental health [46,47,48]. Since avocado consumption is associated with good diet quality, we can hypothesize that regular intake of avocado may mitigate childhood obesity. However, this also requires further investigation.

Our study has some strengths and limitations. A majority of our study participants come from Adventist families that are known to embrace healthy lifestyles. Studies on Adventists show that this group of people have a sizable proportion of vegetarians (~4% vegans and ∼32% lacto-ovo vegetarians) and relatively fewer smokers (~1%) and alcohol drinkers (~7%) [49] compared to the general population. These characteristics then eliminates the need to control for other lifestyle variables that could confound the associations between diet and health and improves our power to find significant associations, if indeed they exist. Our validated food frequency questionnaire was also able to provide the different components needed in the scoring scheme of DQI-I as a measure of diet quality. However, our study sample does not represent the typical US adolescent population, and this limits the generalizability of our findings. About 26% of our sample were vegetarian, which we initially defined as having intake of less than one combined portion per week (approximately 3 oz.) of meat, meat derivatives, poultry, and fish [50], and the majority have highly educated parents. Also, although validated food frequency questionnaires are widely utilized in epidemiological studies, self-reported dietary intake on a food frequency questionnaire is still subject to misreporting bias (either by under-reporting intake of foods not usually eaten, or over-reporting intake of foods known to be socially desirable), particularly in the adolescent population [51]. Also, due to the cross-sectional nature of our study, temporality cannot be assumed. In addition, the low intake of avocado among avocado consumers in our population may have limited our ability to determine the actual relationship between avocado intake and anthropometric health parameters, since there could be a threshold for those desirable effects from a single food. The lack of sufficient intake variation among the avocado consumers also limited our ability to subdivide them into groups that are stable enough for statistical analysis.

Several factors influence the formulation of dietary behaviors and self-directed food choices among the youth [52]. The relatively higher diet quality of our adolescent population compared to reports from other children and adolescent populations may be attributed to home and school influences on their dietary choices and behaviors. However, to what extent these environmental factors affect these behaviors in young people requires further investigation.

## 5. Conclusions

Adolescents who consume avocado exhibit better diet quality and higher intake of plant-based foods and shortfall nutrients compared to adolescents who do not consume avocado. Avocado intake is not associated with measures of obesity and body composition in our adolescent population that come from communities that embrace healthy eating and lifestyle practices. Further studies are needed to determine if there is an optimal amount of avocado that would confer protection from adiposity during adolescence.

## Figures and Tables

**Table 1:** Diet Quality Index—International (DQI-I) components and scoring system *.

DQI-I Component	Scoring Criteria	Scoring
Variety Overall food group intake variety	5 food groups: *Meat*/*poultry*/*fish*/*egg*; *Dairy*/*beans*; *Grains*; *Fruits*; *Vegetables*. Each food group is awarded 0–3 points based on servings eaten per day: ≥1 serving/day (svg/d) = 3 pts; 0 svg/d = 0 pt	**0–20 pts** 0–15
Within-group variety for protein source	6 protein sources: *Meat*, *Poultry*, *Fish*, *Dairy*, *Beans*, *Eggs* ≥3 sources consumed: 5 pts 2 sources consumed: 3 pts. 1 source consumed: 1 pt 0 source consumed: 0 pt	0–5
Adequacy	Adequacy in intake of the following 8 groups based on age-and-gender-specific recommendations: For each of the adequacy groups, 0–5 pts are awarded depending on DGA 2020–2025 recommended svg/d or percentage of RDA met.	**0–40 pts**
	*Vegetables*: ≥2.5 svg/d = 5 pts; 0 svg/d = 0 pt	0–5
	*Fruits*: ≥2 svg/d = 5 pts; 0 svg/d = 0 pt	0–5
	*Grain*: ≥6 oz/d = 5 pts; 0 oz/d = 0 pt	0–5
	*Fiber*: Females: ≥25.0 g/day (g/d) = 5 pts; Males: ≥31 g/d = 5 pts; <0.5/g for Males or Females = 0 pt	0–5
	*Protein*: Females: ≥46.0 g/d = 5 pts; Males: ≥52 g/d = 5 pts; <0.5/g for Males or Females = 0 pt	0–5
	*Iron*: Females: ≥15.0 milligrams/day (mg/d) = 5 pts, <0.3 mg/d = 0; Males: ≥11 mg/d = 5 pts; <0.22 mg/d = 0 pt	0–5
	*Calcium*: ≥1300.0 mg/d = 5 pts; < 26.0 mg = 0 pt	0–5
	*Vitamin C*: Females: ≥65.0 mg/d = 5 pts, <1.3 mg/d = 0; Males: ≥75.0 mg/d = 5 pts; <1.5 mg/d = 0 pt	0–5
Moderation	For each of the 5 moderation groups, 0–6 pts are awarded depending on percentage of RDA met	**0–30 pts**
	*Total fat*: ≤25% of total energy/d = 6 pts; >25–35% of total energy/d = 3 pts; >35% of total energy/d = 0 pt	0–6
	*Saturated fat*:<7% of total energy/d = 6 pts; >7–10% of total energy/d = 3 pts; >10% of total energy/d = 0 pt	0–6
	*Cholesterol*:≤300.0 mg/d = 6 pts; >300–400 mg/d = 3 pts; >400.0 mg/d = 0 pt	0–6
	*Sodium*:≤2300 mg/d = 6 pts; >2300–3300 mg/d = 3 pts; >3300 mg/d = 0 pt	0–6
	*Empty calorie foods*:≤3% total energy/d = 6 pts; >3–10% of total energy = 3 pts; >10% of total energy = 0 pt	0–6
Balance	Balance in the intake of energy from macronutrients ratio is given 0–6 pts, and ratio of the unsaturated to saturated fatty acids is given 0–4 pts.	**0–10 pts**
	*Macronutrient ratio* (*carbohydrate*: *protein*: *fat*): 55.0–65.0: 10.0–15.0: 15.0–25.0 = 6 pts; 52.0–68.0: 9.0–16.0:13.0–27.0 = 4 pts; 50.0–70.0: 8.0–17.0: 12.0–30.0 = 2 pts; else = 0 pt	0–6
	*Fatty acid ratio* (*PUFA*:*MUFA*:*SFA*): both P/S and M/S are 1.0–1.5 = 4 pts; both P/S and M/S are 0.8–1.7 = 2 pts; else = 0 pt	0–4
TOTAL DQI-I	Variety + Adequacy + Moderation + Balance	**0–100 pts**

* Adapted from INDDEX Project, 2018 (https://inddex.nutrition.tufts.edu/data4diets, accessed on 16 July 2021) and Kim et al., 2003 [19]. Italics help distinguish them as variables. pts: points.

**Table 2:** Characteristics of the study population, all participants and according to avocado intake.

Variable	All Participants		Non-Consumer (*n* = 215)		Consumer (*n* = 319)		*p*-Value
	*n* (%)		*n* (%)		*n* (%)		
Gender							0.293 *
Female	304 (56.9)		116 (54.0)		188 (58.9)		
Male	230 (43.1)		99 (46.0)		131 (41.1)		
Site							<0.001 *
California	296 (55.4)		94 (43.7)		202 (63.3)		
Michigan	238 (44.6)		121 (56.3)		117 (36.7)		
Father’s education							0.046
HS or less	98 (18.4)		46 (21.4)		52 (16.3)		
College	206 (38.6)		90 (41.9)		116 (36.4)		
Graduate	230 (43.1)		79 (36.7)		151 (47.3)		
Ethnicity							0.002 *
African/Afr-Am	51 (9.6)		27 (12.6)		24 (7.5)		
Caucasian	209 (39.1)		89 (41.4)		120 (37.6)		
Hispanic	76 (14.2)		15 (7.0)		61 (19.1)		
Asian	60 (11.2)		22 (10.2)		38 (11.9)		
Other	138 (25.8)		62 (28.8)		76 (23.9)		
Dietary pattern							0.413 *
Non-vegetarian	396 (74.2)		164 (76.3)		232 (72.7)		
Vegetarian	138 (25.8)		51 (23.7)		87 (27.3)		
	**Mean**	**SD**	**Mean**	**SD**	**Mean**	**SD**	***p*** **-value**
Age, years	15.0	1.7	14.8	1.8	15.2	1.7	0.010 ^†^
Sleep hours	7.7	1.2	7.9	1.3	7.65	1.21	0.071 ^†^
Energy intake, kcal	2191.0	764.7	2067.1	742.0	2274.52	769.66	0.002 ^†^
	**Median**	**IQR**	**Median**	**IQR**	**Median**	**IQR**	***p*** **-value**
Physical activity, min/d	25.7	12.9, 51.4	25.7	9.6., 41.8	25.7	12.9, 51.4	0.066 ^‡^
Avocado intake, g/d,	4.8	0.0, 8.4	0.0	0.0, 0.0	6.6	5.3, 11.8	<0.001 ^‡^

* Chi-square. † Independent samples *t*-test. ‡ Mann-Whitney U test; SD = standard deviation; IQR = interquartile range; d = day.

**Table 3:** Comparison of Diet Quality Index- International (DQI-I) and its component scores, and anthropometric measures between avocado consumers and non-consumers.

Component *	Score Ranges (Points)	Non-Consumers (*n* = 215)		Consumers (*n* = 319)		*p*-Value
		Mean or Median	SD or IQR	Mean or Median	SD or IQR	
DQI-I total	0–100	62.93	8.45	67.46	7.71	<0.001 ^†^
Variety	0–20	18.04	2.25	18.81	1.54	<0.001 ^‡^
Overall Food Group Variety	0–15	13.57	1.49	14.00	1.18	0.002 ^‡^
Within-group variety for protein source	0–5	4.56	0.92	4.84	0.55	<0.001 ^‡^
Adequacy	0–40	33.41	5.89	36.40	4.08	<0.001 ^‡^
Vegetable group	0–5	3.98	1.21	4.54	0.82	<0.001 ^‡^
Fruit group	0–5	3.21	1.57	4.21	1.17	<0.001 ^‡^
Grain group	0–5	4.62	0.77	4.73	0.64	0.261 ^‡^
Fiber	0–5	3.50	1.19	4.05	1.07	<0.001 ^‡^
Protein	0–5	4.86	0.44	4.96	0.22	0.002 ^‡^
Vitamin C	0–5	4.75	0.69	4.94	0.31	<0.001 ^‡^
Calcium	0–5	3.82	1.15	4.19	0.97	<0.001 ^‡^
Iron	0–5	4.68	0.75	4.78	0.54	0.217 ^‡^
Moderation	0–30	10.17	5.32	10.59	5.75	0.574 ^‡^
Total fat intake	0–6	1.17	1.75	1.52	1.96	0.040 ^‡^
Saturated fat intake	0–6	0.66	1.42	1.23	2.04	0.006 ^‡^
Dietary cholesterol	0–6	5.08	1.99	4.95	2.11	0.409 ^‡^
Sodium intake	0–6	2.22	2.59	1.70	2.37	0.007 ^‡^
Empty calorie foods intake	0–6	1.05	1.25	1.19	1.26	0.088 ^‡^
Balance	0–10	1.31	1.96	1.66	2.24	0.072 ^‡^
Macronutrient ratio (carb:prot:fat)	0–6	0.45	1.20	0.58	1.30	0.097 ^‡^
Fatty acid ratio (PUFA:MUFA:SFA)	0–4	0.87	1.46	1.08	1.54	0.077 ^‡^

* All food and nutrient variables have been energy-adjusted using the residual method. † Two-sample *t*-test; mean (SD) are shown. ‡ Mann-Whitney U test; median [IQR] are shown.

**Table 4:** Comparison * of Dietary Quality Index-International (DQI-I) score and anthropometric parameters between avocado consumers and non-consumers after controlling for confounders.

Variable	Non-Consumer (NC) (*n* = 215)		Consumer (C) (*n* = 319)		C:NC Ratio or EMM Diff		*p*-Value
	EMM	95% CI	EMM	95% CI	EMM	SD or 95% CI	
**DQI-I score**	64.62	63.39, 65.85	68.31	67.19, 69.43	3.69	0.70 ^§^	<0.0001
BMI z-score	0.33	0.16, 0.49	0.28	0.13, 0.43	−0.05	−0.23, 0.13	0.588
Waist-to-Height ratio ^†^	0.46	0.45, 0.47	0.46	0.45, 0.47	1.00 ^‡^	0.98, 1.02	0.855
Fat-free mass ^†^, kg	47.17	46.20, 48.17	46.36	45.46, 47.27	0.98 ^‡^	0.96, 1.01	0.146
Female	40.96	39.83, 42.13	40.83	39.85, 41.84	1.00 ^‡^	0.97, 1.03	0.841
Male	53.50	51.14, 55.97	53.99	51.61, 56.48	1.01 ^‡^	0.96, 1.06	0.720
Fat mass ^†^, kg	10.54	9.56, 11.63	10.52	9.63, 11.49	1.00 ^‡^	0.89, 1.11	0.967
Female	13.95	12.38, 15.71	13.64	12.38, 15.04	0.98 ^‡^	0.85, 1.12	0.752
Male	7.65	6.45, 9.06	8.65	7.30, 10.25	1.13 ^‡^	0.94, 1.37	0.202
% Body fat ^†^	17.61	16.38, 18.92	17.93	16.81, 19.13	1.02 ^‡^	0.94, 1.11	0.659
Female	24.85	22.89, 26.99	24.65	23.09, 26.32	0.99 ^‡^	0.90, 1.09	0.867
Male	12.21	10.73, 13.90	13.44	11.81, 15.29	1.10 ^‡^	0.95, 1.27	0.193

* All comparisons were controlled for age, gender, square term for centered age, study site, father’s educational level (used as surrogate of socio-economic status), ethnicity, sleep hours, physical activity, vegetarian status, total energy (100 kcal/day), and energy-adjusted animal protein intake (10 g/d). Since fat mas, fat-free mass, and % body fat differ between males and females, gender stratification was carried out. † log-transformed to normalize distribution. ‡ ratio of the geometric means of consumers to non-consumers (C:NC ratio) is used in place of difference in estimated marginal means (EMM). § Standard deviation (SD).

**Table 5:** Comparison * of intake of selected food groups and nutrients between avocado consumers and non-consumers.

Food Group/Nutrient	Non-Consumer (NC) (*n* = 215)		Consumer (C) *n* = 319		C:NC Ratio ^‡^ or Mean Diff	95% CI	*p*-Value
	EMM	95% CI	EMM	95% CI			
**FOOD GROUPS, serving/day**							
**Breads, grains, cereals ^†^**	3.9	3.7, 4.1	3.8	3.6, 4.0	0.98	0.93, 1.03	0.456
**Dairy**	2.2	1.9, 2.3	1.9	1.8, 2.1	0.90	0.81, 1.00	0.039
**Animal protein foods ^†^**	0.6	0.4, 0.6	0.5	0.5, 0.6	1.03	0.90, 1.16	0.703
**Plant protein foods^†^**	1.5	1.3, 1.7	1.9	1.7, 2.2	1.31	1.14, 1.51	0.0002
**Fruits ^†^**	1.4	1.2, 1.5	2.0	1.8, 2.2	1.50	1.33, 1.68	<0.0001
**Vegetables ^†^**	2.4	2.3, 2.6	3.1	2.9, 3.3	1.28	1.18, 1.39	<0.0001
**Sweets ^†^**	1.1	1.0, 1.3	1.2	1.1, 1.4	1.10	0.94, 1.30	0.222
**Sweetened beverages ^†^**	0.7	0.6, 0.8	0.8	0.7, 0.9	1.13	0.97, 1.32	0.119
**NUTRIENTS, intake per day**							
**Energy, kcal/d**	2083.0	1961.1, 2204.8	2271.3	2159.9, 2382.7	188.32	51.6, 325.0	0.007
**Total fat, g/d**	76.5	74.2, 78. 8	74.4	72.3, 76.5	−2.10	−4.7, 0.5	0.112
**SFA ^†^, g/d**	24.9	23.8, 26.1	23.0	22.0, 24.0	0.92	0.9, 1.0	0.002
**MUFA, g/d**	25.0	24.2, 25.8	24.9	24.2, 25.7	−0.07	1.0, 0.9	0.875
**PUFA, g/d**	19.4	18.7, 20.1	19.4	18.8, 20.1	0.03	−0.8, 0.8	0.941
**Trans fats, g/d**	2.4	2.3, 2.5	2.2	2.2, 2.3	−0.13	−0.2, −0.0	0.034
**Carbohydrates, g/d**	263.2	257.1, 269.3	266.4	260.8, 272.0	3.22	−3.7, 10.1	0.358
**Protein, g/d**	77.8	75.5, 80.2	80.9	78.8, 83.0	3.09	0.47, 5.71	0.021
**Animal protein ^†^, g/d**	25.7	24.0, 27.5	24.1	22.6, 25.7	0.94	0.87, 1.01	0.112
**Vegetable Protein ^†^, g/d**	43.5	41.4, 45.8	48.4	46.3, 50.7	1.11	1.05, 1.18	0.0002
**Cholesterol ^†^, mg/d**	159.5	148.0, 171.9	149.4	139.5, 160.0	0.94	0.86, 1.02	0.125
**Dietary fiber ^†^, g/d**	23.5	22.5, 24.5	26.3	25.3, 27.3	1.12	1.06, 1.17	<0.0001
**Retinol ^†^, mcg/d**	813.5	773.1, 855.9	875.6	835.8, 917.3	1.08	1.02, 1.14	0.012
**Folate ^†^, mcg/d**	576.1	549.8, 603.7	603.3	578.0, 629.6	1.05	0.99, 1.10	0.086
**Vitamin C ^†^, mg/d**	138.7	127.4, 151.1	164.0	151.8, 177.3	1.18	1.07, 1.30	0.0006
**Vitamin E ^†^, mg/d**	8.8	8.4, 9.2	9.3	8.9, 9.7	1.05	1.00, 1.11	0.070
**Calcium ^†^, mg/d**	1077.3	1032.1, 1124.4	1154.7	1110.3, 1200.8	1.07	1.02, 1.12	0.005
**Magnesium ^†^, mg/d**	330.9	321.4, 340.7	359.5	350.0, 369.1	1.09	1.05, 1.12	<0.0001
**Iron ^†^, mg/d**	17.6	16.9, 18.3	18.67	18.0, 19.3	1.06	1.01, 1.11	0.010
**Zinc, mg/d**	11.6	11.1, 12.0	11.7	11.3, 12.1	0.13	−0.33, 0.59	0.585
**Sodium, mg/d**	3259.1	3167.9, 3350.4	3377.7	3294.2, 3461.1	118.54	16.14, 220.94	0.023
**Potassium ^†^, mg/d**	2671.4	2588.36, 2757.15	2943.55	2859.8, 3029.8	1.10	1.06, 1.14	<0.0001

* All comparisons were controlled for age, gender, site (Michigan or California), educational level of father (high school or less, college, or graduate level), ethnicity (African/African-American, Hispanic, Asian, Caucasian, Other), hours of sleep, moderate-to- vigorous physical activity in mins/d, and vegetarian status (vegetarian or non-vegetarian). † log-transformed to normalize distribution. ‡ ratio of the geometric means of consumers to non-consumers (C:NC ratio) is used in place of difference in estimated marginal means; d = day.

**Table 6:** Demographic and diet quality determinants of avocado intake.

Variable	Estimated β Coefficent	Standard Error	95% CI	*p*-Value
DQI-I: Variety	0.15	0.09	−0.02 to 0.32	0.093
DQI-I: Adequacy	0.11	0.03	0.05 to 0.17	0.0002
DQI-I: Moderation	0.06	0.03	0.01 to 0.11	0.016
DQI-I: Balance	−0.07	0.06	−0.20 to 0.05	0.260
Age	0.12	0.06	−0.01 to 0.25	0.064
Gender				
Female	reference		--	--
Male	−0.28	0.22	−0.70 to 0.15	0.201
Site				
California	reference		--	--
Michigan	−0.46	0.22	−0.89 to −0.04	0.032
Father’s educational level				
High School or less	−0.60	0.29	−1.18 to −0.02	0.042
College	−0.44	0.23	−0.89 to 0.01	0.057
Graduate	reference		--	--
Ethnicity				
Caucasian	reference		--	--
African/Afr-Am	−0.76	0.36	−1.47 to −0.06	0.034
Hispanic	0.82	0.37	0.12 to 1.57	0.025
Asian	−0.25	0.36	−0.95 to 0.46	0.480
Other	−0.30	0.39	−1.07 to 0.47	0.439
Multi-Ethnic	−0.38	0.30	−0.96 to 0.20	0.198
Sleep, hours	−0.15	0.09	−0.33 to 0.02	0.086
Physical activity, mins/day	0.01	0.00	−0.00 to 0.02	0.055
Vegetarian status				
Non-Vegetarian	reference		--	--
Vegetarian	0.21	0.31	−0.39 to 0.83	0.490
